# Progress in the Stereoselective Synthesis Methods of Pyrrolidine-Containing Drugs and Their Precursors

**DOI:** 10.3390/ijms252011158

**Published:** 2024-10-17

**Authors:** Andrey Smolobochkin, Almir Gazizov, Nurbol Appazov, Oleg Sinyashin, Alexander Burilov

**Affiliations:** 1 Arbuzov Institute of Organic and Physical Chemistry, FRC Kazan Scientific Center, Russian Academy of Sciences, Arbuzov Str., 8, Kazan 420088, Russia; agazizov@iopc.ru (A.G.); oleg@iopc.ru (O.S.); burilov@iopc.ru (A.B.); 2 Laboratory of Engineering Profile, Department of Engineering Technology, Korkyt Ata Kyzylorda University, Aiteke bi Str., 29A, Kyzylorda 120014, Kazakhstan

**Keywords:** pyrrolidine, precursors, drugs

## Abstract

The presented review systematizes and summarizes the data on the synthesis of pyrrolidine derivatives, which are precursors for obtaining drugs. Based on the analysis of published data, the most promising directions in the synthesis of biologically active compounds containing a pyrrolidine ring are identified. Stereoselective synthesis methods are classified based on the source of the pyrrolidine ring. The first group includes methods that use a pyrrolidine ring as the starting compound. The second group combines stereoselective methods of cyclization of acyclic starting compounds, which lead to optically pure pyrrolidine derivatives.

## 1. Introduction

Nitrogen-containing heterocyclic compounds have received considerable attention because of their biological and pharmacological significance [1,2,3,4,5]. The pyrrolidine fragment is part of the structure of many alkaloids [6], such as nicotine, preussin, hygrine, ruspolinone, cuscohygrine, etc., which have a wide range of biological activity. In addition, pyrrolidine derivatives are precursors in the synthesis of tropane alkaloids (tropane, atropine, cocaine, etc.) [7,8]. Vitamin B_12_ and its varieties can also be noted [9,10,11].

In addition, pyrrolidine derivatives are found in virtually every living organism in the form of the proline amino acid, which is an essential building block of peptides and proteins [12,13]. The proline fragment is part of many natural peptide hormones [14], such as oxytocin, α-melanocyte-stimulating hormone, vasopressin, gastrin, angiotensin, insulin, adrenocorticotropic hormone, β-endorphin, bradykinin, calcitonin, motilin, peptide YY, thyrotropin-releasing hormone, cholecystokinin, exenatide, erythropoietin, pramlintide, etc.

**Figure 1:**
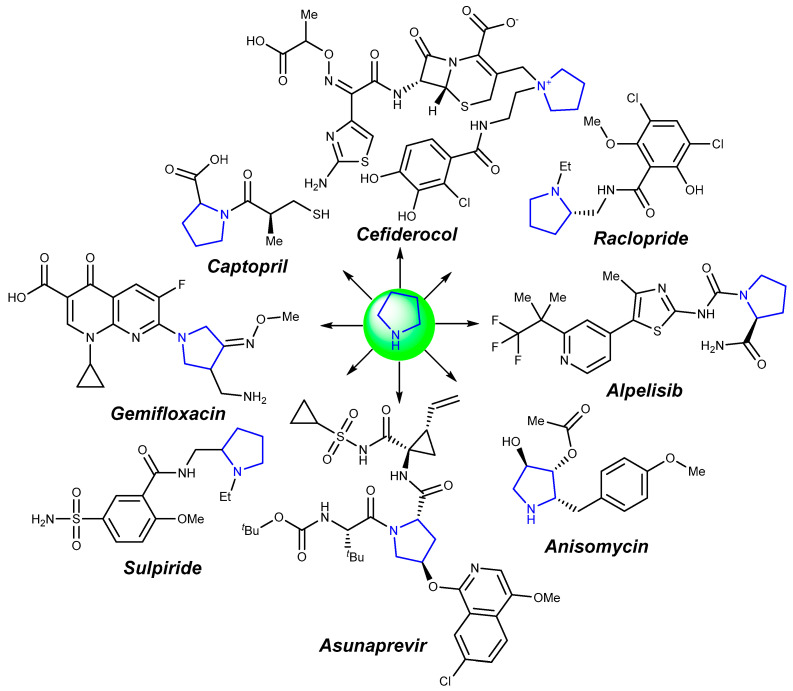
Pyrrolidine-based drugs.

Thus, the pyrrolidine ring acts as an important building block of natural compounds: vitamins, hormones, and alkaloids, as well as synthetic drugs. It should also be noted that pyrrolidine derivatives are one of the most common heterocyclic fragments in drugs [15]. Examples include Captopril, Cefiderocol, Raclopride, Alpelisib, Anisomycin, Asunaprevir, Sulpiride, Gemifloxacin, etc. These are only a small part of the pyrrolidine-containing drugs. The synthesis of some drugs will be reported in this article (Figure 1). Therefore, stereoselective synthesis of pyrrolidine derivatives, which are precursors in drug synthesis, is currently of great interest and represents a serious problem in synthetic organic chemistry.

This review does not discuss the introduction of protective groups into amino acid molecules, in particular proline, which is often the starting compound for the synthesis of drugs, since there are several reviews on this topic [16,17]. In addition, this review does not consider methods for the synthesis of amino acid amides, which are quite widely known [18,19,20].

A systematic analysis of the methods for synthesizing precursors of pyrrolidine-containing drugs has not been performed to date. There are just a few reviews that describe methods for obtaining either specific drugs or a group of drugs with similar pharmacological activity. Thus, in 2016, a monograph by Berestovitskaya B.M. et al. was published [21], which describes in sufficient detail the methods for obtaining racetams (piracetam, oxiracetam, seletracetam, levetiracetam, fasoracetam, fonturacetam, aniracetam, pramiracetam, coluracetam, brivaracetam, methylphenylpiracetam, nebracetam, nefiracetam, omberacetam). Racetams are a large class of low-molecular-weight psychoactive drugs containing the pyrrolidone ring that are widely used in the treatment of various disorders such as epilepsy, dementia, depression, anxiety, and hypoxia [22,23,24,25]. A review [26] provides a detailed discussion of the chemistry of dactinomycin. There is a review of pyrrolidine derivatives with anticancer activity [27,28], Pyrrolidine Derivatives as Anti-diabetic Agents [29].

This review summarizes stereoselective methods for the synthesis of pyrrolidine derivatives, which are building blocks of drugs. The methods can be divided into two classes based on the source of the pyrrolidine ring. In the first case, a pyrrolidine fragment is used, the source of which is usually proline or 4-hydroxyproline. In this case, the functionalization of the ready optically pure cyclic source is carried out. In the second case, the source of pyrrolidine is acyclic starting compounds.

## 2. Stereoselective Methods for the Synthesis of Substituted Pyrrolidines from Cyclic Precursors

### 2.1. Synthesis of Substituted Pyrrolidines from Proline and 4-Hydroxyproline Derivatives

**Scheme 1:**
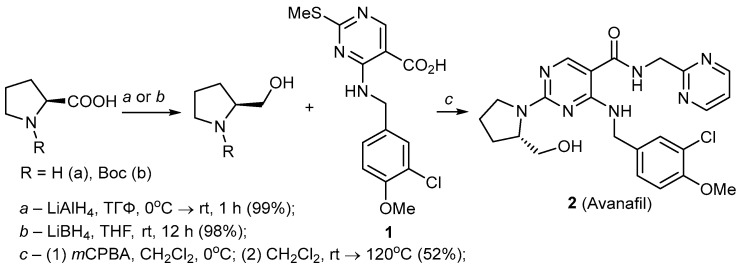
Synthesis of Avanafil.

Pyrrolidine-containing drugs are predominantly synthesized from cyclic precursors. As a rule, drug synthesis begins from proline, 4-hydroxyproline, and their derivatives. Thus, (*S*)-prolinol is the starting compound for the synthesis of many drugs. The main method of preparation is the reduction of proline using LiAlH_4_ [30,31,32,33,34,35,36] or LiBH_4_ [37,38]. Further condensation of carboxylic acid **1** with prolinole leads to Avanafil **2** [39,40,41], known under the trademarks “Stendra” and “Spedra” (Scheme 1). The drug is used for the treatment of erectile dysfunction. Avanafil inhibits phosphodiesterase-5, preventing the degradation of cGMP [42].

**Scheme 2:**
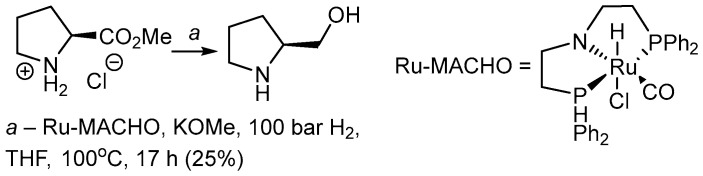
Synthesis of (*S*)-pyrrolidin-2-ylmethanol.

There are other methods for the synthesis of prolinol, which are less common. In 2017, Harald Gröger and co-workers [43] reported the Ru-catalyzed hydrogenation of pyrrolidine with only 25% yield (Scheme 2).

**Scheme 3:**
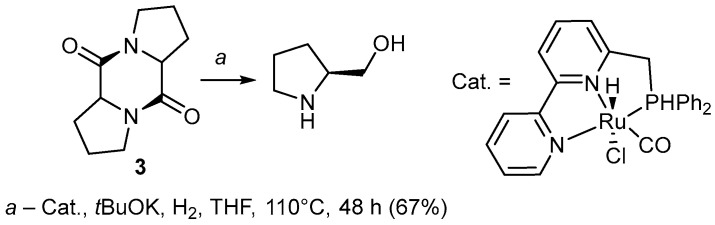
Synthesis of (*S*)-pyrrolidin-2-ylmethanol.

Reductive opening of the piperazine ring of compound **3** in an autoclave under a hydrogen atmosphere in the presence of a Ru catalyst leads to prolinol in 67% yield (Scheme 3) [44].

**Scheme 4:**
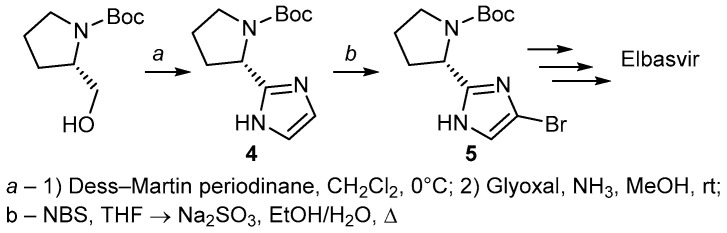
Synthesis of precursor Elbasvir.

Elbasvir selective NS5A inhibitor of the hepatitis C virus NS5A replication complex [45,46]. It should be noted that side effects have only been assessed in the combination with Grazoprevir. At one of the stages of the synthesis of Elbasvir, used for the treatment of hepatitis C, (S)-*tert*-butyl 2-(4-bromo-1*H*-imidazol-2-yl)pyrrolidin-1-carboxylate **4** is used [47,48]. In this case the 4-bromimidazole derivative **4** is obtained by oxidation of (*S*)-prolinol with Dess–Martin periodinane followed by condensation with glyoxal in the presence of ammonia in methanol (Scheme 4). It is noteworthy that the aldehyde formed during the oxidation of alcohol does not have to be isolated. Bromination of imidazole with *N*-bromosuccinimide (NBS) followed by treatment with Na_2_SO_3_ leads to bromide **5** [45,49,50,51].

It should be noted that prolinol can also be oxidized while maintaining the chiral center in a mixture of (2,2,6,6-tetramethylpiperidin-1-yl)oxyl with sodium perchlorate [52,53], SO_3_ complex pyridine [54], with oxolyl chloride in DMSO (Swern oxidation) [55].

**Scheme 5:**
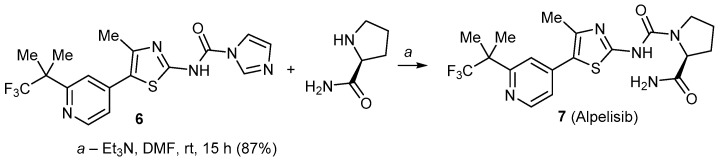
Synthesis of Alpelisib.

In 2019, the drug Alpelisib was approved for the treatment of certain types of breast cancer. It is an α-specific PI3K inhibitor that selectively inhibits p110α [56,57]. Alpelisib **7** is obtained by reacting the pyridine derivative **6** with L-prolinamide (Scheme 5). It should be noted that the product yield is high, exceeding 80% [58,59].

**Scheme 6:**
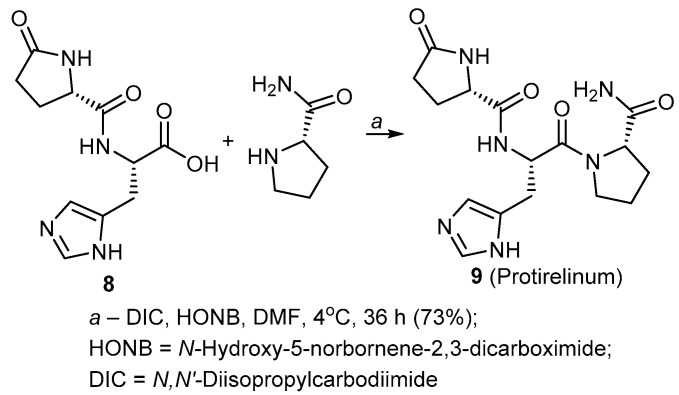
Synthesis of Protirelin.

In 1969, R. Guillemin and A. Schally [60,61] determined the structure of and synthesized Protirelin (thyrotropin-releasing hormone) **9**, which is the smallest known peptide hormone successfully used to treat epilepsy in children. It is also used as a diagnostic test for the thyroid gland disease [62,63,64]. For this discovery, they were awarded the Nobel Prize in 1977. Protirelin **9** was obtained by the reaction of acid **8** with (*S*)-pyrrolidine-2-carboxamide (Scheme 6) [65,66,67].

**Scheme 7:**
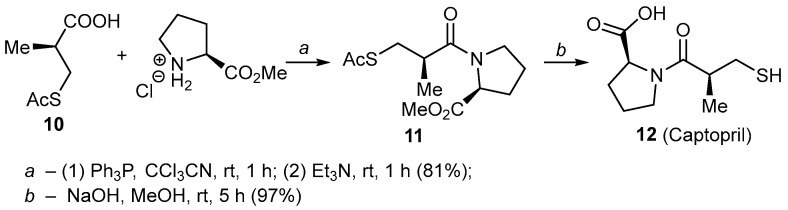
Synthesis of Captopril.

A method for the synthesis of Captopril is presented. It is an angiotensin-converting enzyme (ACE) inhibitor [68,69], based on a two-stage reaction of (*S*)-3-(acetylthio)-2-methylpropanoic acid (**10**) with trichloroacetonitrile and hydrochloric L-proline methyl ester. Hydrolysis of amide **11** produces Captopril **12** (Scheme 7).

**Scheme 8:**
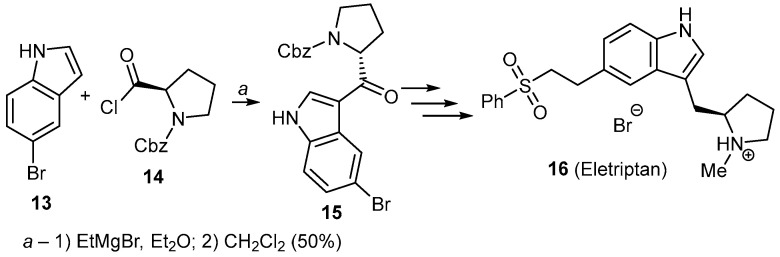
Synthesis of Eletriptan.

Another pyrrolidine-containing drug is Eletriptan **16** (trade name Relpax) [70,71,72], which is used to treat migraines. Eletriptan reduces swelling of the blood vessels surrounding the brain. This swelling is associated with the head pain of a migraine. The synthesis of Eletriptan **16** [73,74] is based on the reaction of 5-bromo-1*H*-indole (**13**) with ethylmagnesium bromide followed by the addition of pyrrolidine **14**, which leads to compound **15**. After several stages, it is possible to isolate Eletriptan hydrobromide (Scheme 8).

**Scheme 9:**
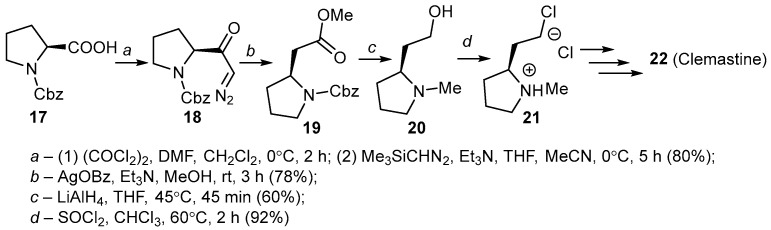
Synthesis of precursor Clemastine.

An original synthetic method was proposed [75,76] for the preparation of pyrrolidine **21**, which is a precursor in the synthesis of Clemastine **22**, used to relieve the symptoms of hay fever and allergies [77,78]. The key step of the approach is the Arndt–Eistert reaction, consisting in the conversion of carboxylic acid **17** into the closest homologue **19** using trimethylsilyldiazomethane. Reduction of the ester and Cbz-deprotection with lithium aluminum hydride in THF results in a hydroxyethylpyrrolidine derivative. Chlorination with thionyl chloride made it possible to obtain a chloroethylpyrrolidine derivative **21** (Scheme 9).

**Scheme 10:**
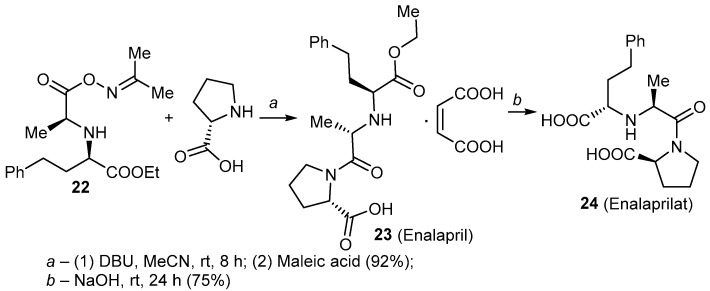
Synthesis of Enalapril and Enalaprilat.

Condensation of compound **22** with proline in acetonitrile at room temperature leads to Enalapril **23**, which was isolated as the maleic acid salt in 92% yield (Scheme 10) [79]. Treatment of Enalapril with NaOH [80,81] leads to Enalaprilat **24**, used for the treatment of arterial hypertension [82,83]. It is on the World Health Organization’s List of Essential Medicines.

Raclopride **27** is a selective dopamine antagonist that is widely used in positron emission tomography (PET) studies for the diagnosis of movement disorders (first mentioned [84] in 1985) [85,86,87]. It is produced through multistep syntheses. Pyrrolidin-2-carboxamide is reduced with lithium aluminum hydride to pyrrolidin-2-ylmethanamine [88,89,90]. It is noteworthy that this method can be used to reduce both the *S*- and *R*-isomers of pyrrolidine-2-carboxamide while maintaining optical purity.

**Scheme 11:**
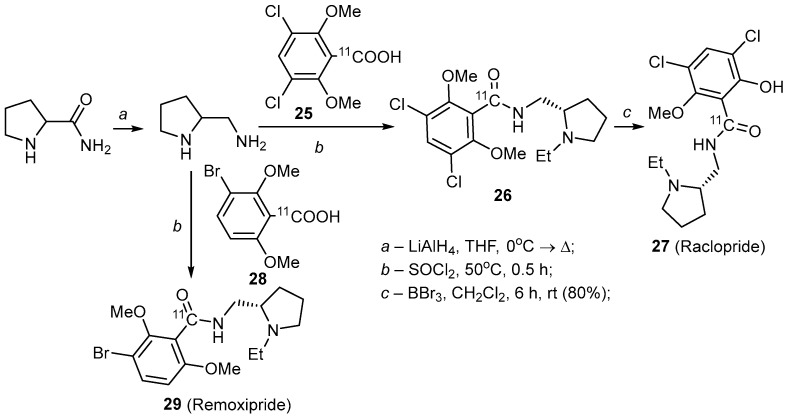
Synthesis of Raclopride and Remoxypride.

Next, the amine reacts with 2,6-dimethoxybenzoic acid (**25**) containing the ^11^C isotope to form acid, which reacts with thionyl chloride to form an acid chloride. The reaction of the latter with (*S*)-(1-ethylpyrrolidin-2-yl)methanamine leads to the pyrrolidine derivative **21**, which reacts with BBr_3_ to form Raclopride **27** (Scheme 11) [91,92,93,94,95,96,97]. Remoxypride **29** can also be synthesized from amine pyrrolidin-2-ylmethanamine as a result of the reaction of acid **28** in the presence of thionyl chloride [91,92,94,98]. Remoxypride [99,100,101] is an antipsychotic drug that was previously used to treat schizophrenia but was withdrawn from clinical practice due to high toxicity.

**Scheme 12:**
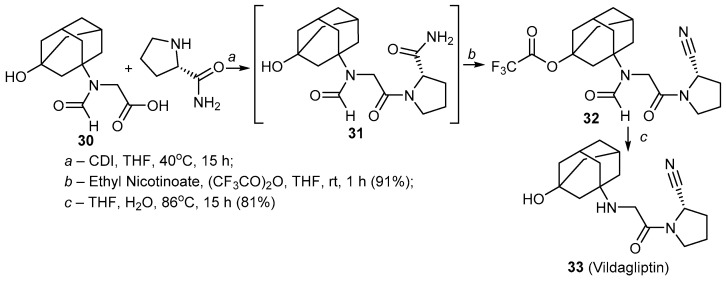
Synthesis of Vildagliptin.

The study [102] describes the synthesis of Vildagliptin **33** [103,104,105,106], a drug for the treatment of type 2 diabetes mellitus (of the dipeptidyl peptidase-4 inhibitor class of drugs). Reaction of acid **30** with L-prolinamide in the presence of *N*,*N’*-carbonyldiimidazole (CDI) leads to intermediate amide **31**, which, after treatment with ethyl nicotinate in trifluoroacetic anhydride, leads to nitrile **32**. In the last step of the targeted synthesis, all protecting groups were removed using aqueous trifluoroacetic acid, which made it possible to obtain Vildagliptin **33** in good yield (Scheme 12).

**Scheme 13:**
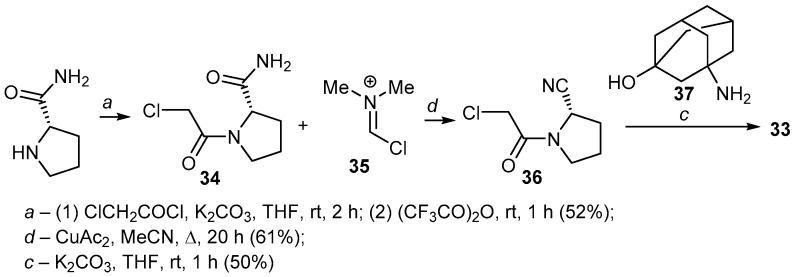
Synthesis of Vildagliptin.

The reaction of pyrrolidine **34** with Vilsmeyer’s reagent **35** yielded nitrile **36** while maintaining the configuration of the chiral center. The subsequent reaction of the resulting compound with amine **37** also made it possible to isolate Vildagliptin **33** (Scheme 13) [43,107,108].

**Scheme 14:**
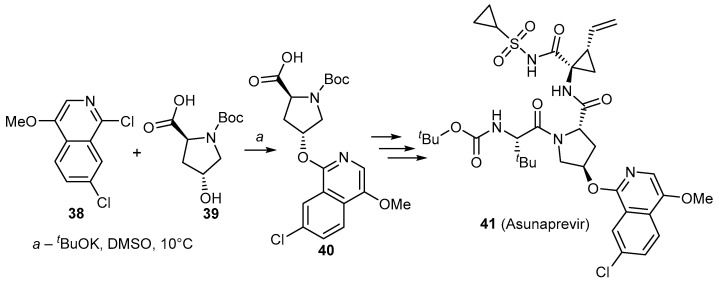
Synthesis of precursor Asunaprevir.

Antiviral drugs are medicines intended to treat various viral diseases. It is very interesting that all antiviral drugs containing a pyrrolidine ring are used for the treatment of hepatitis C. Typically they are an inhibitor of hepatitis C virus enzyme serine protease NS3 [109]. Thus, the key stage in the synthesis of Asunaprevir **41** (trade name “Sunvepra”) [110,111] is the Williamson reaction between 1-chloroisoquinoline **38** and the 4-hydroxyproline derivative **39** in the presence of *tert*-potassium butoxide, resulting in the formation of ether **40** (Scheme 14) [112].

**Scheme 15:**
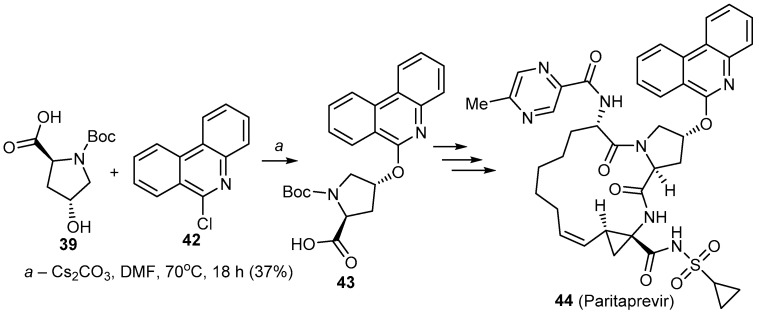
Synthesis of precursor Paritaprevir.

Paritaprevir (ABT-450) **44** is used as a component of combination drugs [113,114]. The pyrrolidine ring is introduced by the Williamson reaction between 3-hydroxy-L-proline **39** and 6-chlorophenanthridine **42** in the presence of cesium carbonate in DMF under heating (Scheme 15) [115]. The resulting compound undergoes a series of transformations, which makes it possible to obtain Paritaprevir **44**.

**Scheme 16:**
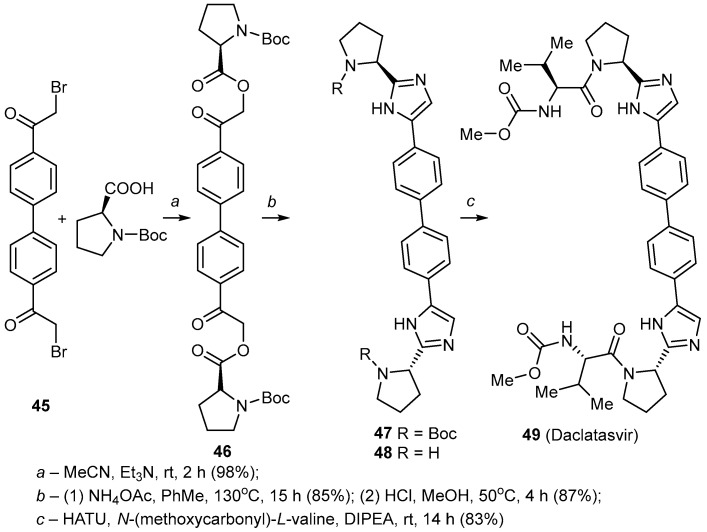
Synthesis of Daclatasvir.

A series of studies [116,117,118] describes the multi-stage synthesis of Daclatasvir **49** [119,120], which is often used in combination with other drugs. It should be noted that the World Health Organization included the drug Daclatasvir in the list of the most essential and important drugs in the healthcare system. The synthesis of this drug is based on the initial alkylation of *N*-protected proline with 1,1′-([1,1′-biphenyl]-4,4′-diyl)bis(2-bromoethanonone) (**45**), which leads to the bis-ketoester **46**. Compound **46**, in turn, undergoes amidation to form bis-imidazole **47**. Further deprotection under acid catalysis results in bis-pyrrolidine **48**, which reacts with *N*-(methoxycarbonyl)-L-valine to form the target product **49** (Scheme 16).

**Scheme 17:**
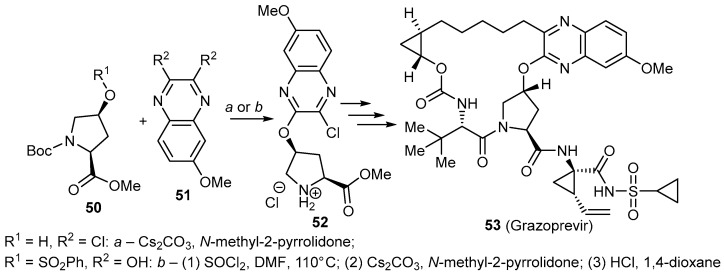
Synthesis of precursor Grazoprevir.

Grazoprevir **53** [121,122] is often used in combination with Elbasvir or Ribavirin [123,124,125]. The method for the synthesis of this compound is based on the reaction of the pyrrolidine derivative **50** with 2,3-dichloro-6-methoxyquinoxaline **51** in the presence of cesium carbonate (method a) [126,127]. The use of 6-methoxyquinoxaline-2,3-diol [121] (method b) also made it possible to isolate the target compound **52** in high yield (Scheme 17).

**Scheme 18:**
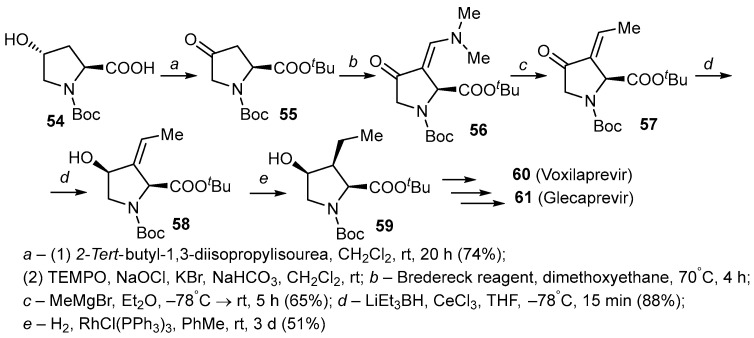
Synthesis of precursor Voxilaprevir and Glecaprevir.

Voxilaprevir **60** is used in combination with Sofosbuvir and Velpatasvir, which has the tradename Vosevi [128,129]. Pyrrolidine **59** is used as the starting compound for Voxilaprevir synthesis. Pyrrolidine derivative **59** is also the basic structure for the synthesis [130] of Glecaprevir **61 [131,132]** (Scheme 18). The synthesis starts with the commercially available *Boc*-protected *trans*-4-hydroxy-L-proline **54**, which is first esterified with 2-*tert*-butyl-1,3-diisopropylisourea and then converted to the corresponding ketoproline **55** by TEMPO oxidation. The preparation of enaminone **56** was carried out using the Bredereck reagent (*tert*-butoxybis(dimethylamino)methane) followed by reaction with the Grignard reagent to obtain enone **57**. Reduction of **57** with lithium triethylborohydride provides high stereoselectivity and the best yield of alcohol **58**. Hydrogenation was carried out in the presence of a Wilkinson catalyst. The reaction proceeded slowly but stereoselectively, allowing the isolation of the only diastereomer **59** [133,134].

**Scheme 19:**
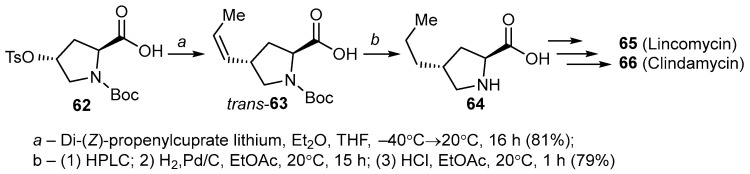
Synthesis of precursor Lincomycin and Lincomycin.

In the 1960s, Lincomycin **65**, an antibiotic that is used to treat severe infectious and inflammatory diseases, in particular sepsis, osteomyelitis, septic endocarditis, pneumonia, etc., began to be introduced into clinical practice [135,136,137]. However, because of its adverse effects and toxicity, it is rarely used today and is reserved for patients allergic to penicillin. Lincomycin is produced by the actinomycete *Streptomyces lincolnensis* [138,139]; it can also be obtained as a result of a multi-stage synthesis based on proline derivative **64**. Thus, the tosylate **62** is alkylated with an excess of lithium di-(Z)-propenylcuprate to obtain a mixture of *cis*- and *trans*-4-propenylproline (1:2.2 ratio), which cannot be separated by conventional methods. It should be noted that the isomerization of the double bond was insignificant and did not exceed 4–10%. Diastereomers were separated by preparative HPLC. Next, the multiple bonds of the *trans*-**63** isomer were subjected to reduction; at the last stage, the protecting group was removed in the presence of hydrochloric acid, which made it possible to isolate product **64** in good yield (Scheme 19) [140]. A derivative of Lincomycin, Clindamycin **66**, which is used to treat a number of bacterial infections [141,142], is obtained by chlorination of lincomycin [143].

**Scheme 20:**
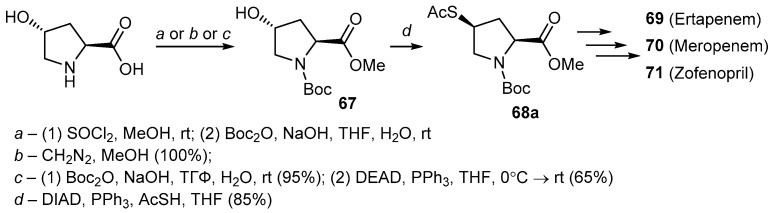
Synthesis of precursor Ertapenem, Meropenem and Zofenopril.

A large group of antibiotics is carbapenems [144,145], which kill bacteria by binding to penicillin-binding proteins, thus inhibiting bacterial cell wall synthesis. A representative of this class of antibiotics is Ertapenem **69**, intended for the treatment of complicated intra-abdominal infections and community-acquired pneumonia [146,147,148,149]. The pyrrolidine fragment is part of the synthetic broad-spectrum antibiotic Meropenem **70 [150,151,152,153]** and Zofenopril **71** [154,155,156], which protects the heart and helps reduce high blood pressure. The synthesis of Ertapenem and Meropenem was carried out on the basis of (2*S*,4*R*)-4-hydroxypyrrolidine carboxylic acid (Scheme 20). The authors of papers [157,158,159,160,161] described a method for the synthesis of ester **67** by treatment with thionyl chloride in methanol followed by the introduction of a protecting *Boc*-group (method *a*). Ester **68a** can be obtained with quantitative yield by treating the amino acid with diazomethane in methanol (method b) [162,163]. Ester **68a** was isolated in moderate yield through the Mitsunobu reaction (method *c*) [164,165,166]. The pyrrolidin-3-ylethanethioate derivative **68a** can also be obtained as a result of the Mitsunobu reaction between 4 –hydroxypyrrolidine **67** and thioacetic acid (method *d*) [167].

**Scheme 21:**
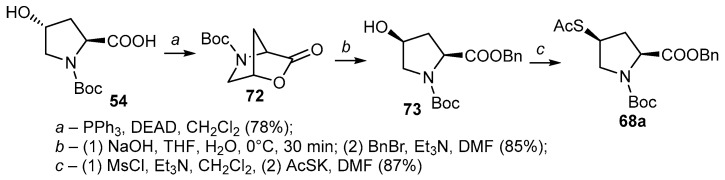
Synthesis of pyrrolidin-3-ylethanethanioate derivative.

The intramolecular Mitsunobu reaction of compound **54** followed by treatment with sodium hydroxide and benzyl bromide led to the *cis*-4-hydroxyproline derivative **73** (Scheme 21). Further, with the use of methanesulfonyl chloride (MsCl) and potassium thioacetate, it was possible to isolate the pyrrolidin-3-ylethanethanioate derivative **68a** [168,169,170].

**Scheme 22:**
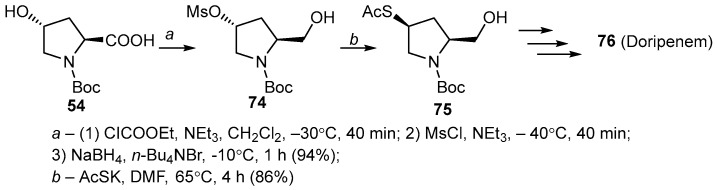
Synthesis of (*S*)-pyrrolidin-2-ylmethanol derivative.

Doripenem **76** also belongs to Carbapenem class antibiotics [171,172,173]. It should be noted that this drug can fight Pseudomonas aeruginosa, which is resistant to many antibiotics. The synthesis of Doripenem is reported in the papers [174,175,176]. Specifically, 4-hydroxyproline **54** was converted to mesylate **74** in 94% yield as a result of several reactions: formation of a mixed anhydride with ethyl chloroformate, mesylation with methanesulfonyl chloride, and reduction of the mixed anhydride with sodium borohydride. Mesylate **74** was treated with potassium thioacetate in DMF to obtain thioacetate **75** with an inversion of the configuration of the chiral center at the fourth carbon atom (Scheme 22).

**Scheme 23:**
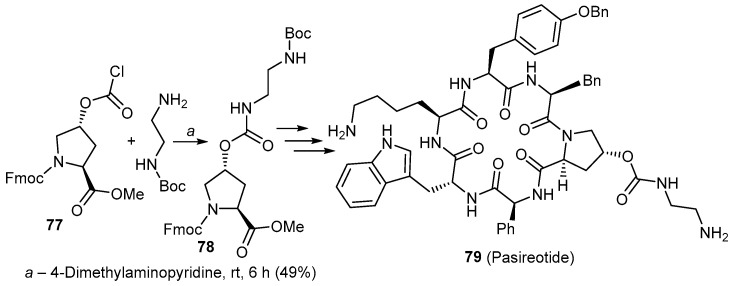
Synthesis of precursor Pasireotide.

The synthetic cyclic hexapeptide Pasireotide **79** is used in the treatment of Cushing’s disease [177,178,179]. Pasireotide is a somatostatin analog with an increased affinity to somatostatin receptor 5. It is prepared by reacting pyrrolidine **77** with *Boc*-diaminoethane in the presence of 4-dimethylaminopyridine (DMAP), and product **78** was isolated with 98% purity through a series of steps (Scheme 23) [180,181].

The method for obtaining pyrrolidine-containing drugs presented in this section, which consists in modifying natural proline and 4-hydroxyproline, allows one to obtain products with high optical purity. However, the disadvantage of this method is the inability to easily introduce pharmacophores into various positions of the pyrrolidine fragment, which complicates the search for potentially biologically active compounds.

### 2.2. Semi-Synthetic and Peptide Drugs Containing a Pyrrolidine Ring

We will not consider in detail the methods for the synthesis of semi-synthetic drugs, since a fragment of proline or 4-hydroxyproline is already included in the intermediate product. A large group of drugs is represented by antifungal drugs from the class of Echinocandins [182,183,184,185]—macrocycles of natural origin, or their semisynthetic derivatives. Thus, Micafungin [186,187,188,189], an antifungal drug of the echinocandin group, can be synthesized from the peptide FR901379 produced by *Coleophoma empetri* [190]. The drug Caspofungin [191,192,193] is obtained from the natural product Pneumocandin B_0_, the producer of which is the fungus *Glarea lozoyensis* [194]. Anifungin [195,196,197] is obtained from Echinocandin B, which is a metabolic product of the fungus *Aspergillus nidulans* Var. *echinulatu A 32204*.

**Figure 2:**
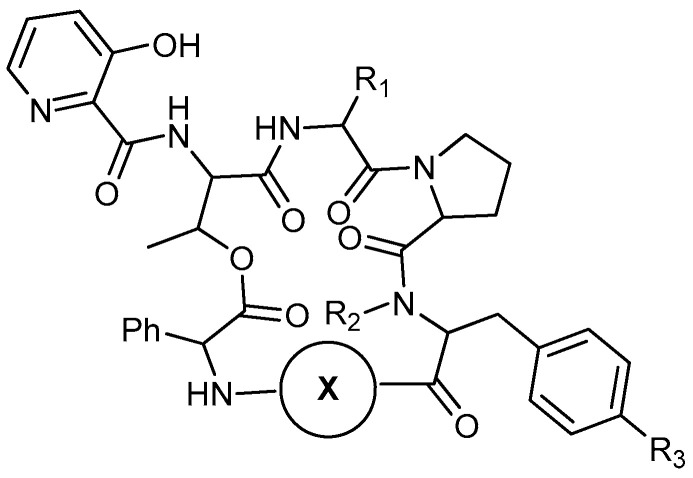
Streptogramin antibiotics.

A large group of streptogramin antibiotics (Figure 2) should be noted, containing a proline fragment, known for almost 50 years. Streptogramins (Streptogramin B, Mikamycin B, Synergistin B, Ostreogrycin B, Pristinamycin, Vernamycin B (β, γ, δ), Vernamycin C, Virginiamycin S (1–5), patricin A, patricin B, Quinupristin) are potent drugs against numerous highly resistant pathogens and are therefore used as potent antibiotics. Streptogramins act as inhibitors of bacterial protein synthesis. These natural products are cyclic hexa- or hepta-peptides produced by various representatives of the *Streptomyces* bacteria genus [198,199,200,201,202]. There is a monograph on the chemistry of streptogramin preparations [203], which describes antibiotics of this group in some detail, including their synthesis, and we refer interested readers to this work.

Pyrrolidine-containing hormones are predominantly represented by peptides and polypeptides, which contain the amino acid proline or hydroxyproline. A typical representative of this class of hormones is gonadotropin-releasing hormone (gonadorelin, gonadoliberin, gonadotropin-releasing factor), which has been widely used in clinical medicine since it was identified and synthesized in 1971 [204]. Gonadotropin-releasing hormone is one of the representatives of the class of releasing hormones of the hypothalamus [205]. Analogs of gonadorelin are nafarelin, histrelin, decapeptyl, leuprorelin, buserelin, and deslorelin [206,207,208]; its antagonists are degarelix, ganirelix, abarelix, antarelix, and cetrorelix [209,210], and its agonists are goserelin and triptorelin [211].

Here, we do not describe the preparation of peptide drugs, since they are synthesized in the same way as most peptides, including using solid-phase peptide synthesis methods. We will only list some representatives who have recently entered medical practice. Leuprorelin is a synthetic hormone used to treat prostate cancer, breast cancer, endometriosis, and uterine fibroids [212,213]. Bivalirudin [214,215] is a direct thrombin inhibitor often used as an anticoagulant in invasive cardiology (used since 2000).

Icatibant [216,217,218], marketed under the brand name Firazyr (2008), is a drug for the symptomatic treatment of acute attacks of hereditary angioedema. Ganirelix is a synthetic peptide that acts as a gonadotropin-releasing hormone antagonist and is used as a drug to treat infertility [219,220]. Linaclotide (2012) is used to treat constipation [221,222,223].

Despite the apparent simplicity of obtaining drugs from this group, this is currently a popular method for obtaining new drugs, especially antibiotics. Over time, antibacterial drugs lose their effectiveness due to acquired bacterial resistance. Modification of the basic structure leads to a new drug that can fight bacteria.

### 2.3. Synthesis of Pyrrolidine Derivatives from Other Cyclic Precursors

**Scheme 24:**
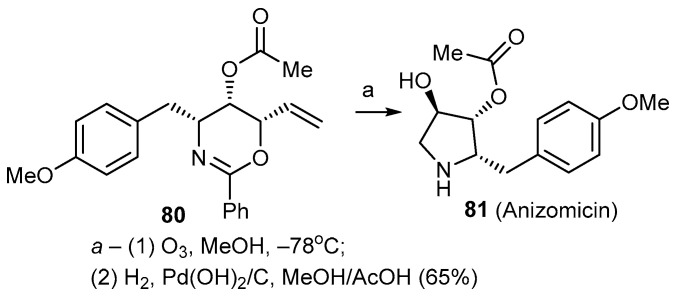
Syntheses of Anisomycin.

This section, like the previous one, is devoted to a description of the synthesis of pyrrolyline derivatives based on cyclic precursors. Thus, the stereoselective method for the synthesis of Anisomycin **81** from oxazine **80** includes ozonolysis with heterocycle opening and subsequent intramolecular cyclization of the resulting aminoaldehyde to the pyrrolidine derivative **81** (Scheme 24). The reaction is carried out in a MeOH/AcOH mixture in the presence of Pd(OH)_2_ at room temperature [224]. Anisomycin **81** is an antibiotic first isolated by Sobin and Tanner in 1954 from the *Streptomyces griseolus* and *Streptomyces roseochromogenes* bacteria [225]; however, its absolute configuration was finally established only in 1968 [226,227]. Anisomycin interferes with protein and DNA synthesis by inhibiting peptidyl transferase or the 80S ribosome system. In addition, Anisomycin is used as a component of Martin Lewis agar [228].

**Scheme 25:**
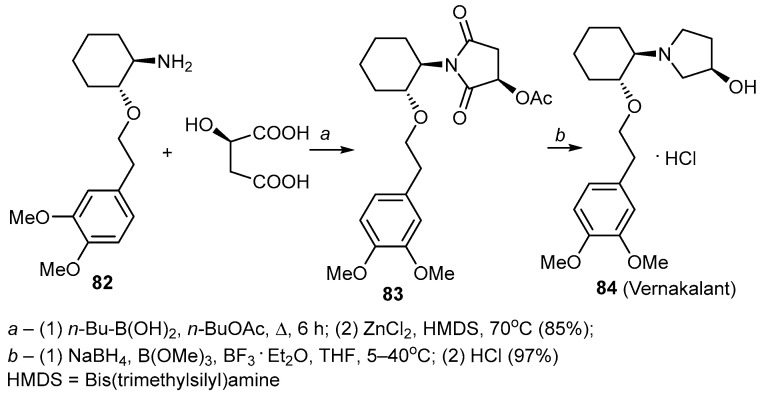
Syntheses of Vernakalant.

Succinimide **83** can also be prepared by intermolecular cyclization of amine **82** and (*R*)-2-hydroxysuccinic acid (Scheme 25). After reduction of compound **83**, Vernakalant **84** was isolated as a hydrochloride with a yield of 97% and a purity of 99.5% [229]. Vernakalant was developed as an antiarrhythmic drug intended for rapid conversion of atrial fibrillation to sinus rhythm. It acts in higher heart rates.

**Scheme 26:**
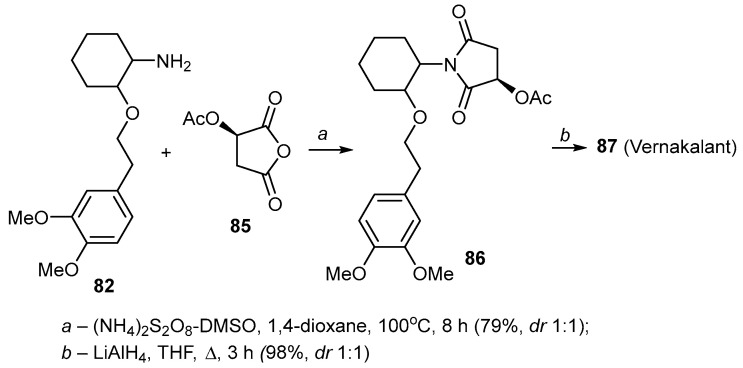
Syntheses of Vernakalant.

A study [230] proposed a method for the synthesis of the antiarrhythmic drug Vernakalant **87** [231,232,233,234,235]. The interaction of (*R*)-acetoxyacetic anhydride **85** with amine **82** leads to succinimide 86, the reduction of which allows one to obtain the target compound **87** (Scheme 26). The product is formed as a 1:1 mixture of diastereomers, which the authors were unable to separate, making the method unsuitable for drug synthesis.

**Scheme 27:**
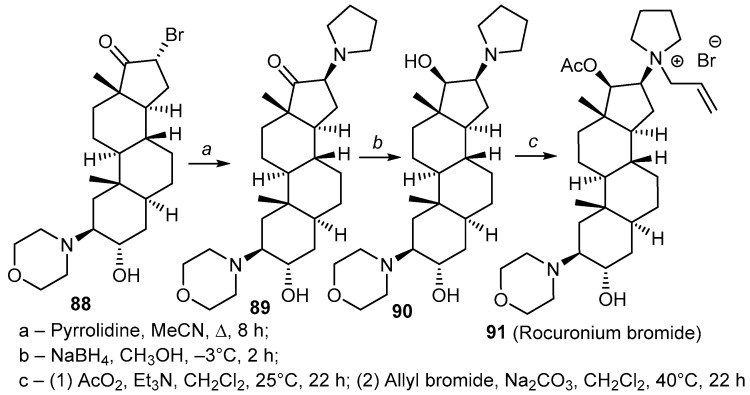
Syntheses of Rocuronium bromide.

Since 1994 [236], the muscle relaxant rocuronium bromide **91** has been used in clinical practice (in surgery) [236,237]. The synthesis of rocuronium bromide **91** involves the condensation of steroid **88** and pyrrolidine in boiling acetonitrile with inversion of the stereocenter. Next, ketone **89** is reduced to alcohol **90**, followed by acylation and reaction with allyl bromide (Scheme 27) [238,239].

**Scheme 28:**
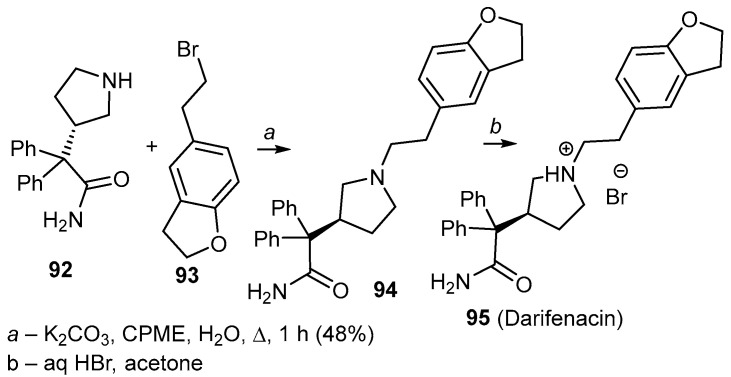
Syntheses of Darifenacin.

Darifenacin **95**, approved by the FDA in 2004, is used to treat urinary incontinence [240,241]. It works by blocking the M3 muscarinic acetylcholine receptor, which is responsible for bladder muscle contractions. An efficient process for the preparation of Darifenacin hydrobromide **95**, based on the reaction of amide **92** with bromo-alkane **93**, is demonstrated [242] (Scheme 28). After recrystallization, the purity of the product was >99.7%. It should be noted that the authors chose cyclopentyl methyl ether (CPME) as a solvent because it has a number of advantages: high boiling point and high resistance to acids/alkalis. In addition, CPME serves as both a reaction solvent and an extraction solvent, thereby avoiding the need for solvent removal.

Derivatives of oxazine, pyrrolidine-2,5-dione, and pyrrolidine itself are not widely used for drug synthesis, since it is necessary to obtain optically pure starting compounds, which is not required in the case of proline. Therefore, the data presented in this section are largely of a fundamental value.

## 3. Stereoselective Methods for the Synthesis of Pyrrolidine Derivatives from Acyclic Precursors

**Scheme 29:**
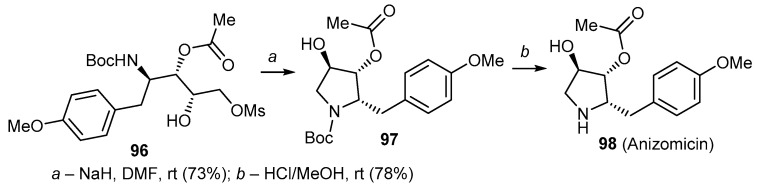
Syntheses of Anisomycin.

The material presented in this section is fundamentally different from that in the previous ones. Thus, the formation of a pyrrolidine skeleton occurs as a result of cyclization of acyclic compounds, and this process can be realized both intra- and intermolecularly. The papers [243,244] describe a convenient enantioselective approach to the synthesis of Anisomycin, the key step of which is the cyclization of alcohol **96** with the use of NaH in DMF at room temperature to give *Boc*-protected pyrrolidine **97**. Removal of the protecting group in an acidic environment leads to Anisomycin **98** in 78% yield (Scheme 29).

**Scheme 30:**
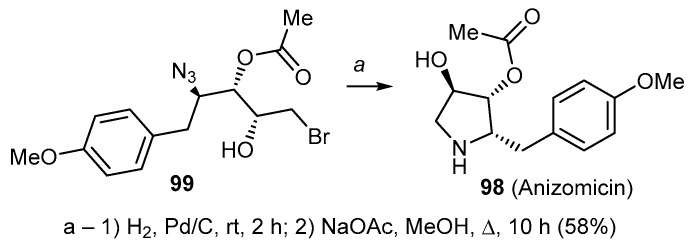
Syntheses of Anisomycin.

Using a similar scheme, the authors of works [245,246] carried out the cyclization of azide **99** in two stages. Initially, in the presence of palladium on carbon, the azide group was reduced to amine. Further intramolecular cyclization of the amine occurs upon reflux in the presence of NaOAc in MeOH and leads to the formation of Anisomycin **98** with a yield of 58% (Scheme 30).

**Scheme 31:**
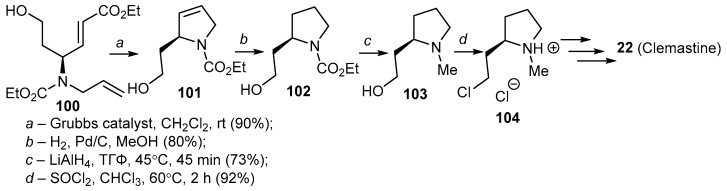
Synthesis of precursor Clemastine.

Cyclization of an alkene **100** in methylene chloride in the presence of a Grubbs catalyst leads to a 2-pyrroline derivative **101** (*ee* = 95%), hydrogenation of which over palladium on carbon leads to pyrrolidine **102** (Scheme 31). Removal of the ester group with lithium aluminum hydride results in an alcohol **103,** from which the target product is obtained in good yield [247,248]. It is noteworthy that the starting compound **104** for the synthesis of Clemastine **22** is obtained in the same number of steps and with comparable yield as from the Cbz-protected prolinen **21** described in Section 2.1. [75,76] and from acyclic amide **100** [247,248].

**Scheme 32:**
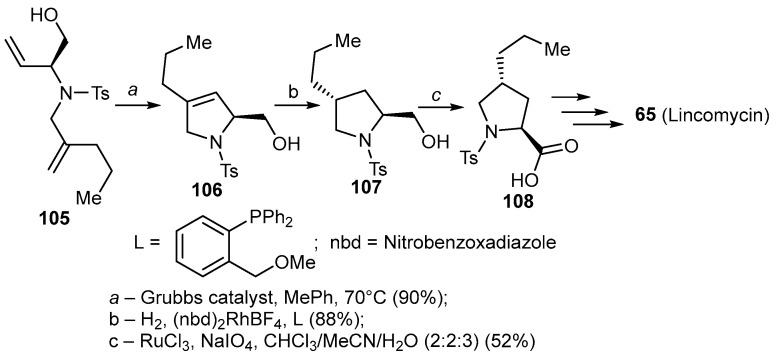
Synthesis of precursor Lincomycin.

R.W. Bates et al. [249] described the synthesis of the acid **108**. The method is based on the intramolecular cyclization of an alkene **105** to a 2-pyrroline derivative **106**, the hydrogenation of which produces an alcohol **107**. By oxidizing the latter with ruthenium tetroxide, obtained in situ under the conditions of the Sharpless reaction, the acid is obtained. It should be noted that, in contrast to the method for the synthesis of Lincomycin **65** described in Section 2.1 [140], this method is characterized by a greater number of steps and a lower yield of product (Scheme 32).

**Scheme 33:**
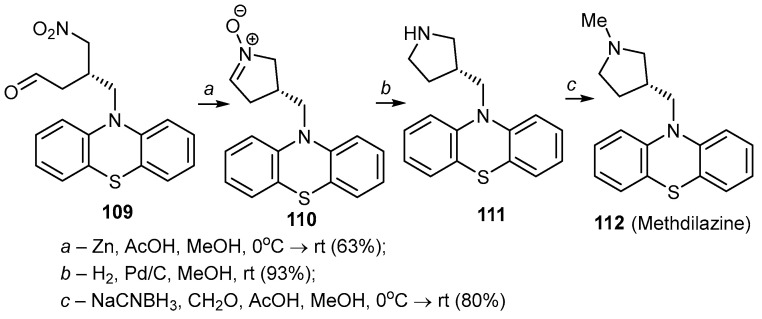
Syntheses of Metdilazine.

The antihistamine drug Metdilazine **112** [250,251,252] is synthesized in several stages: treatment of aldehyde **109** with zinc powder in a mixture of acetic acid and methanol leads to nitrone **110**, which is reduced to pyrrolidine **111**. After treatment of compound **111** with formaldehyde in the presence of NaCNBH_3_ and acetic acid in methanol, as well as recrystallization from ethanol, the yield of product **112** was 80% (Scheme 33) [253].

**Scheme 34:**
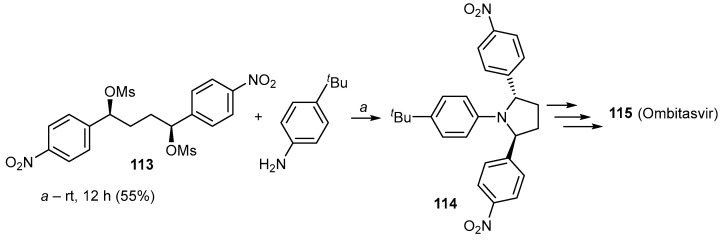
Synthesis of pyrrolidine derivative.

At the end of 2014, Ombitasvir **115** [254,255] was approved by the FDA for the treatment of hepatitis C as part of combination drugs. Since 2018, this drug has been included in the list of Vital and Essential Medicines (VEDs). The starting compound is pyrrolidine **114**, the synthesis of which is based on the reaction of dimesylate **113** with aniline (Scheme 34) [256,257].

Despite the few examples, the data on the cyclization of starting acyclic compounds presented in this section indicate the high potential of this approach. First of all, it is the absence of dependence on proline as a basic structure, which will make it possible to obtain pyrrolidines containing the necessary substituent/substituents at any position of the cycle. This method also allows one to obtain a wide range of compounds, which will greatly simplify the screening of potential drugs.

## 4. Conclusions

An analysis of the literature data on the synthesis of pharmacological drugs indicates that the pyrrolidine skeleton is part of many vital drugs. One of the largest groups of drugs containing a pyrrolidine moiety is antiviral drugs. It should be noted here that it is this group that leads in the number of drugs approved for use in the last five years. We emphasize that this development is in no way connected with the spread of the SARS-CoV-2 coronavirus—these drugs were introduced into clinical practice in 2014–2019. The pandemic that began in 2019 has only further demonstrated the urgent need for affordable antiviral drugs.

Less attention is paid to cardiovascular, neurotropic, and antihistamine drugs. It is noteworthy that all of these drugs, unlike those mentioned above, are low-molecular-weight derivatives of pyrrolidine. Thus, there is a clear tendency to complicate the structure of pyrrolidine-containing drugs—structurally relatively simple compounds are giving way to polyfunctional and often polycyclic polypeptide molecules containing several chiral centers.

Approaches to the synthesis of precursors of pyrrolidine-containing drugs can be reduced to several main types, with separate mention being made of drugs of natural origin and their semi-synthetic derivatives, the structure of which initially includes the pyrrolidine ring, as well as various polypeptides obtained by classical methods of peptide synthesis. The most common method for the synthesis of intermediates of most drugs containing a pyrrolidine ring is the introduction of a ready-made heterocycle into the molecule, often containing a pre-formed chiral center. This ensures the production of optically pure compounds with good product yield. Proline, 4-hydroxyproline, or their various derivatives are widely used to introduce a pyrrolidine fragment. Less common is the introduction of unsubstituted pyrrolidine. Formation of the pyrrolidine ring from acyclic precursors is even less common. This is probably due to the requirement of obtaining most drugs in the form of individual stereoisomers, which imposes serious limitations on both the enantioselectivity of these reactions and the methods for isolating target compounds. However, this approach also has a number of advantages in the future, the most important of which is the possibility of introducing additional pharmacophore groups into the pyrrolidine ring at the stage of its formation, which opens up prospects for the creation of new types of pyrrolidine-containing drugs. The data presented in this review indicate that such studies are currently actively developing and are in high demand in modern organic and medicinal chemistry.

## List of Acronyms

The following acronyms were used in the review:

Boc	*tert*-butoxycarbonyl,
Cbz	benzyloxycarbonyl,
CDI	1,1′-carbonyldiimidazole,
DBU	1,8-diazabicyclo [5.4.0]undec-7-ene,
DEAD	diethyl azodicarboxylate,
DIAD	Diisopropyl azodicarboxylate
DIPEA	diisopropylethylamine,
HATU	Hexafluorophosphate azabenzotriazole tetramethyl uronium
